# A GC–MS-based untargeted metabolomics approach for comprehensive metabolic profiling of mycophenolate mofetil-induced toxicity in mice

**DOI:** 10.3389/fmolb.2024.1332090

**Published:** 2024-03-07

**Authors:** Tongfeng Zhao, Yaxin Zhao, Haotian Chen, Wenxue Sun, Yun Guan

**Affiliations:** ^1^ Department of Hematology, Jining No.1 People’s Hospital, Jining, China; ^2^ Department of Pharmacy, Jining No.1 People’s Hospital, Jining, China; ^3^ Translational Pharmaceutical Laboratory, Jining No.1 People’s Hospital, Jining, China; ^4^ Postdoctoral of Shandong University of Traditional Chinese Medicine, Jinan, China

**Keywords:** mycophenolate mofetil, toxicity, gas chromatography-mass spectrometry, multiparameter flow cytometry analysis, lymphocytes

## Abstract

**Background:** Mycophenolate mofetil (MMF), the morpholinoethyl ester of mycophenolic acid, is widely used for maintenance immunosuppression in transplantation. The gastrointestinal toxicity of MMF has been widely uncovered. However, the comprehensive metabolic analysis of MMF-induced toxicity is lacking. This study is aimed to ascertain the metabolic changes after MMF administration in mice.

**Methods:** A total of 700 mg MMF was dissolved in 7 mL dimethyl sulfoxide (DMSO), and then 0.5 mL of mixture was diluted with 4.5 mL of saline (100 mg/kg). Mice in the treatment group (*n* = 9) were given MMF (0.1 mL/10 g) each day via intraperitoneal injection lasting for 2 weeks, while those in the control group (*n* = 9) received the same amount of blank solvent (DMSO: saline = 1:9). Gas chromatography-mass spectrometry was utilized to identify the metabolic profiling in serum samples and multiple organ tissues of mice. The potential metabolites were identified using orthogonal partial least squares discrimination analysis. Meanwhile, we used the MetaboAnalyst 5.0 (http://www.metaboanalyst.ca) and Kyoto Encyclopedia of Genes and Genomes database (http://www.kegg.jp) to depict the metabolic pathways. The percentages of lymphocytes in spleens were assessed by multiparameter flow cytometry analysis.

**Results:** Compared to the control group, we observed that MMF treatment induced differential expression of metabolites in the intestine, hippocampus, lung, liver, kidney, heart, serum, and cortex tissues. Subsequently, we demonstrated that multiple amino acids metabolism and fatty acids biosynthesis were disrupted following MMF treatment. Additionally, MMF challenge dramatically increased CD4^+^ T cell percentages but had no significant influences on other types of lymphocytes.

**Conclusion:** MMF can affect the metabolism in various organs and serum in mice. These data may provide preliminary judgement for MMF-induced toxicity and understand the metabolic mechanism of MMF more comprehensively.

## Introduction

Suppression of the immune system is crucial after organ transplantation and is usually administrated for the treatment of a variety of autoimmune diseases. Mycophenolate mofetil (MMF), the morpholinoethyl ester of mycophenolic acid (MPA), is widely used for maintenance immunosuppression in solid organ, bone marrow, and stem cell transplantation (Srinivas et al., 2003a; Moiseev et al., 2016a; Carlone et al., 2021a). MMF also serves as a substitution for cyclosporine A therapy-induced severe nephrotoxicity or hemolytic uremic syndrome in renal transplant recipients (Ulinski et al., 2005). MMF exerts inhibitory effect on the proliferation of both B- and T-lymphocytes via non-competitively and reversibly blocks the *de novo* synthesis of guanine nucleotides required for DNA and RNA synthesis during lymphocyte proliferation (Wu, 1994). Unfortunately, it has been proved that the application of immunosuppressant is associated with the occurrence of hematologic toxicity or other organ toxicity.

The gastrointestinal (GI) toxicity is the most common adverse effects of MMF including diarrhea, abdominal pain, nausea, and vomiting, which result in dose reduction or drug withdrawal, and the increased risk of rejection and death after transplantation (Calmet et al., 2015). It has been reported that friability on endoscopy was associated with severe disease; and nausea and erythema were related to poor prognosis (Bhattacharya et al., 2022). Recently, a study demonstrated an intact intestinal microbiota was essential to initiate and sustain the MMF-induced GI toxicity, and MMF exposure was associated with alterations of intestinal flora composition, companied the increase in genes involved in lipopolysaccharide (LPS) biosynthesis (Flannigan et al., 2018). However, the underlying mechanism of MMF-induced GI toxicity remains unclarified. Meanwhile, the administration of MMF is more frequently associated with hematologic toxicity, such as anemia, due to bone marrow suppression or hemolysis, leukopenia, and thrombocytopenia (Danesi and Del Tacca, 2004). Neutropenia and leukopenia have been reported as severe hematologic toxicities associated with MMF treatment in transplant recipients (Nogueras et al., 2005; Varnell et al., 2017). In addition, uncommon side effect, such as hepatotoxicity, is also observed in renal transplant recipients (Balal et al., 2005), and a recent study has proved that MMF-induced hepatotoxicity is associated with mitochondrial abnormality in liver transplant recipients and mice (Warren et al., 2021). Therefore, it is paramount to investigate the mechanisms responsible for MMF-associated toxicity.

Metabolomics approach is widely implemented in systems biology research and it can be systematically used to identify potential metabolic biomarkers under the condition of stimulation or impact. In this study, a GC-MS-based untargeted metabolomics approach was used to investigate the underlying mechanism of MMF-associated toxicity. Alterations in metabolites of serum and other organs were identified, and disrupted metabolic pathways were analyzed to elucidate the metabolic profiling after MMF exposure. This study represents the first comprehensive evaluation of metabolic profiling in MMF-treated mice.

## Materials and methods

### Chemicals and reagents

MMF with purity of 99%, heptadecanoic acid (purity ≥ 98%), methanol (chromatographic grade), and pyridine were purchased from Macklin Biochemical (Shanghai, China). O-methylhydroxylamine hydrochloride (purity ≥ 98%) was obtained from J&K Scientific Ltd. (Beijing, China). N, O-bis(trimethylsilyl)trifluoroacetamide (containing 1% trimethylchlorosilane) was obtained from Sigma Aldrich (St. Louis, MO, United States). The purified water was purchased from Wahaha (Hangzhou, China). RPMI medium was obtained from (Thermo Fisher Scientific, Waltham, MA, United States).

### Animals and treatment

A total of 18 C57BL/6 mice weighting 35 ± 5 g (6-week-old) were purchased from Pengyue Experimental Animal Breeding Co., Ltd. (Jinan, China). Animals were housed under a 12 h light/dark cycle at 20°C–22°C. All experimental procedures conformed to the Guidelines for the Use of Laboratory Animals, and approved by the Ethical Committee for Animal Experimentation of Jining First People’s Hospital (Approval No. JNRM-2023-DW-021).

A total of 700 mg MMF was dissolved in 7 mL dimethyl sulfoxide (DMSO), and then 0.5 mL of mixture was diluted with 4.5 mL of saline (100 mg/kg). After 1-week of acclimation, mice in MMF group (*n* = 9) were intraperitoneally injected with MMF (0.1 mL/10g) every day for 2 weeks, and the dose was adjusted with the increase in body weight. Mice received the same amount of blank solvent (DMSO: saline = 1:9) served as control group.

### Sample collection

Mice were anesthetized with 1% sodium pentobarbital (50 mg/kg) after the last administration in mice. Blood samples were collected from each mouse after eyeball enucleation, centrifuged at 4,000 rpm for 10 min at 4°C, and serum samples were then stored at −80°C. Subsequently, mice were sacrificed by cervical dislocation and at the same time, the intestine, hippocampus, lung, liver, kidney, heart, cortex and spleen tissues were immediately collected on ice, frozen in liquid nitrogen, and then stored at −80°C for succeeding experiments.

### Sample preparation

A 100-μL aliquot of sample was mixed with 350 μL of heptadecanoic acid (100 μg/mL in methanol). After centrifugation at 14,000 rpm for 15 min at 4°C, the supernatant liquid was dried with liquid nitrogen at 37°C. Subsequently, 80 μL of O-methylhydroxylamine hydrochloride (15 mg/mL in pyridine) was added and incubated at 70°C for 90 min. A total of 100 μL of N, O-bis(trimethylsilyl)trifluoroacetamide containing 1% trimethyl chlorosilane was added to each sample, followed by incubation at 70°C for 60 min.

Tissue sample (50 mg for each) was homogenized with 1 mL of methanol, mixed with 50 μL of heptadecanoic acid (1 mg/mL in methanol), and centrifuged at 14,000 rpm for 15 min at 4°C. O-methylhydroxylamine hydrochloride (80 μL; 15 mg/mL in pyridine) was then added at 70°C for 90 min, mixed with 100 μL of N, O-bis(trimethylsilyl)trifluoroacetamide (containing 1% trimethyl chlorosilane), and incubated at 70°C for 60 min. Pooling 10 µL of each sample of control group and MMF group served as quality control (QC). A 0.22-μm filter was used to purify samples for further GC-MS analysis.

### Lymphocytes isolation

The excised spleens were placed in RPMI medium. For the preparation of single cell suspensions, spleen tissues were firstly ground, passed through a 70-mm-pore mesh, and washed with RPMI. After that, the samples were centrifuged (350 × g) at 4°C for 10 min, followed by abandoning the supernatants. In order to lyse the erythrocytes, the cells were resuspended in 3 mL of TRIS-ammonium chloride solution (0.144 M of NH_4_Cl plus 0.017 M of TRIS; pH 7.2), incubated at 4°C for 2 min, and then washed with RPMI for twice. After centrifugation (350 × g at 4°C for 10 min), the cells were re-suspended in RPMI containing 10% FBS and lymphocytes from spleen were obtained.

### Multiparameter flow cytometry analysis

Multiparameter flow cytometry was performed according to a standard protocol. Single-cell suspensions were incubated with anti-CD16/32 monoclonal antibody (Biolegend, San Diego, CA, United States) for 15 min at room temperature to block Fc receptors before staining with the specific antibodies. Spleen mononuclear cells were then stained with the indicated fluorescent monoclonal antibodies for surface molecules. Intracellular staining was performed using fixation and permeabilization buffers (eBioscience) according to the manufacturers’ instructions.

### GC-MS based metabolomics analysis

An Agilent 7890B GC system coupled to a 7000C GC/MS Triple Quad Mass Detector (Agilent Technologies, United States), equipped with an HP-5MS fused silica capillary column (30 m × 0.25 mm × 0.25 μm), was implemented for metabolomics analysis. Helium was used as the carrier gas with a flow rate at 1 mL/min. Sample (1 μL) was injected into GC-MS with a split ratio of 50:1. The injection temperature was set to 280°C, transfer line temperature was 250°C, and ion source temperature was 230°C, respectively. Electron collision ionization was set to −70 EV, and the frequency of acquisition was 20 spectra/s. The ionization mode of mass spectrometry is electrospray ionization with a mass/charge (m/z) full scan range of 50–800.

We have successfully uploaded the source data from our GC-MS analysis to MetaboLights, a move aimed at enhancing transparency and simplifying access to the raw data for the scientific community. Our research project is exclusively identified by the code MTBLS9315. To delve into our study, you can follow this link: https://www.ebi.ac.uk/metabolights/MTBLS9315.

### Data processing and multivariate analysis

Raw data from GC-MS was analyzed using the Agilent Mass Hunter (Version B.07.00, Agilent Technologies, CA, United States). The preprocessing included alignment, retention time correction, baseline filtration, and deconvolution. For metabolite identification, a library containing all QC samples was established, and the U.S. National Institute of Standards and Technology (NIST 14) GC-MS library was applied to identify the unknown metabolites of QC samples. The metabolites with similarity > 80% were considered as structurally identified (Caterino et al., 2020; Zhang et al., 2021). Subsequently, a new spectrum library named “New Library” was constructed, and used for spectrum matching of metabolites of experimental samples. Manually validation was conducted to reduce deconvolution errors in the process of automatic data processing and to eliminate false identifications.

An integrated data matrix composed of the peak index (RT-m/z pair), sample name, and corresponding peak area was obtained. Peak area was normalized using Microsoft Excel™ (Microsoft, Redmond, WA, United States). SIMCA-P 14.0 (Umetrics, Sartorius Stedim Biotech) was implemented to further analyze the data by multivariate analysis including principal components analysis (PCA) and orthogonal partial least squares discrimination analysis (OPLS-DA). The two-tailed Student’s *t*-tests were carried out to compare differences of the two groups. Compounds with variable importance in projection (VIP) values > 1.0 and two-tailed Student’s t-test *p* values < 0.05 were considered as potential differential expressed metabolites. Clustered heatmap diagram and bubble diagram (pathway enrichment) were constructed using MetaboAnalyst 5.0 (http://www.metaboanalyst.ca).

## Results

### GC–MS total ion chromatograms (TICs) of samples

**FIGURE 1:**
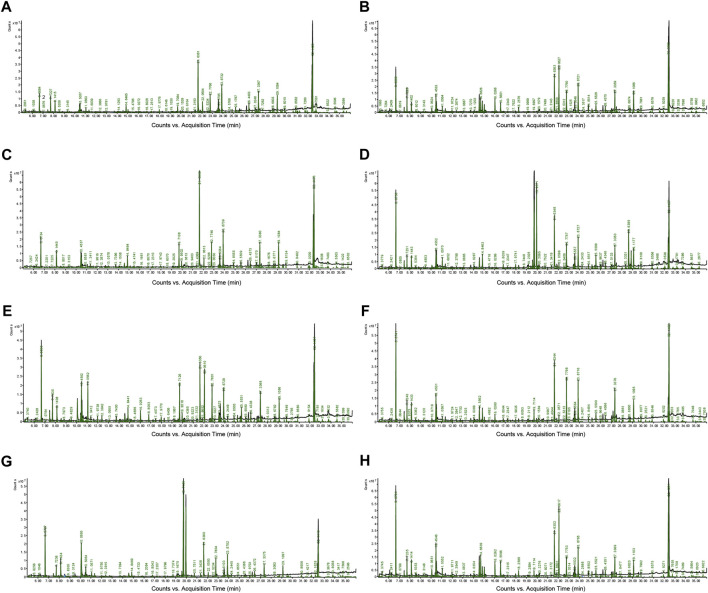
Representative gas chromatography–mass spectrometry (GC–MS) total ion chromatograms (TICs) of quality control (QC). **(A)** Intestine **(B)** Hippocampus **(C)** Lung **(D)** Liver **(E)** Kidney **(F)** Heart **(G)** Serum **(H)** Cortex.

As illustrated in Figure 1, representative GC-MS TICs of QC from tissue and serum samples are presented. Supplementary Tables S1–S8 includes retention time, metabolites, % similarity, molecular formula, CAS number, peak area, and molecular weight for different tissues and serum. The QC in our study were prepared by pooling 10 µL of each sample from the control group and MMF group. We indicated that there were significant differences in TICs among different QC samples. Supplementary Figure S1 showed that there was a linear positive correlation between retention time and retention index curve.

### Metabolomic data analysis

**FIGURE 2:**
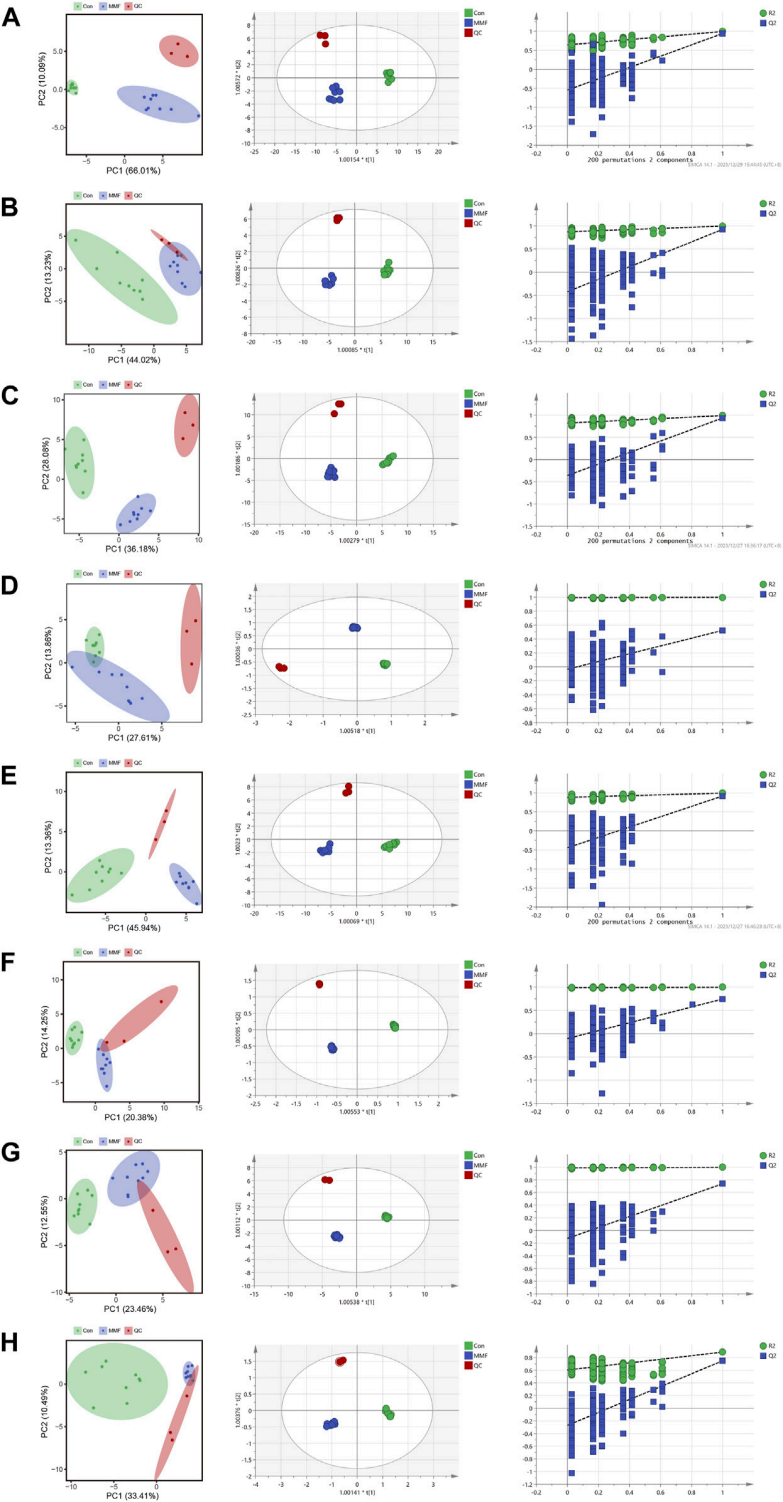
PCA analysis, OPLS-DA score plots and 200 permutation tests. **(A)** Intestine **(B)** Hippocampus **(C)** Lung **(D)** Liver **(E)** Kidney **(F)** Heart **(G)** Serum **(H)** Cortex.

PCA was used to distinguish the metabolic profiles. As illustrated in Figures 2A–H, clear differences between the control and the MMF groups were observed in intestine, hippocampus, lung, liver, kidney, heart, serum and cortex samples. In PCA model, the total variance explained was 76.1%, 57.3%, 64.3%, 41.5%, 59.3%, 34.6%, 36.0%, and 43.9% respectively, indicating satisfactory construct validity. The parameter scores of OPLS-DA were shown in Table 1. Meanwhile, a ranking test was conducted to verify the validity of OPLS-DA model. The abscissa represents the predicted principal component score of the first principal component, the ordinate represents the orthogonal principal component score, and the color represents different experimental groups. In different tissues and serum, the proportion of variance explained by the OPLS-DA model (R^2^X) was 72.8%, 51.5%, 53.9%, 42.3%, 60.1%, 42.6%, 36.1%, and 43.7%, respectively. We found that the intersection points between blue regression line (Q^2^-point) and vertical axis were all negative values (Figures 2A–H). These results confirmed the validity of OPLS-DA model and at the same time, the significantly differences across the MMF and control groups were validated.

**TABLE 1:** OPLS-DA parameter scores.

Tissues	R^2^X (cum)	R^2^Y (cum)	Q^2^ (cum)
Intestine	0.728	0.971	0.862
Hippocampus	0.515	0.978	0.836
Lung	0.539	0.984	0.895
Liver	0.423	0.998	0.513
Kidney	0.601	0.987	0.842
Heart	0.426	0.998	0.52
Serum	0.361	0.997	0.546
Cortex	0.437	0.992	0.595

R^2^X: the explanation rate of the X matrices; R^2^Y: the explanation rate of the Y matrices; Q^2^: the prediction ability.

### Identification of differential metabolites

**FIGURE 3:**
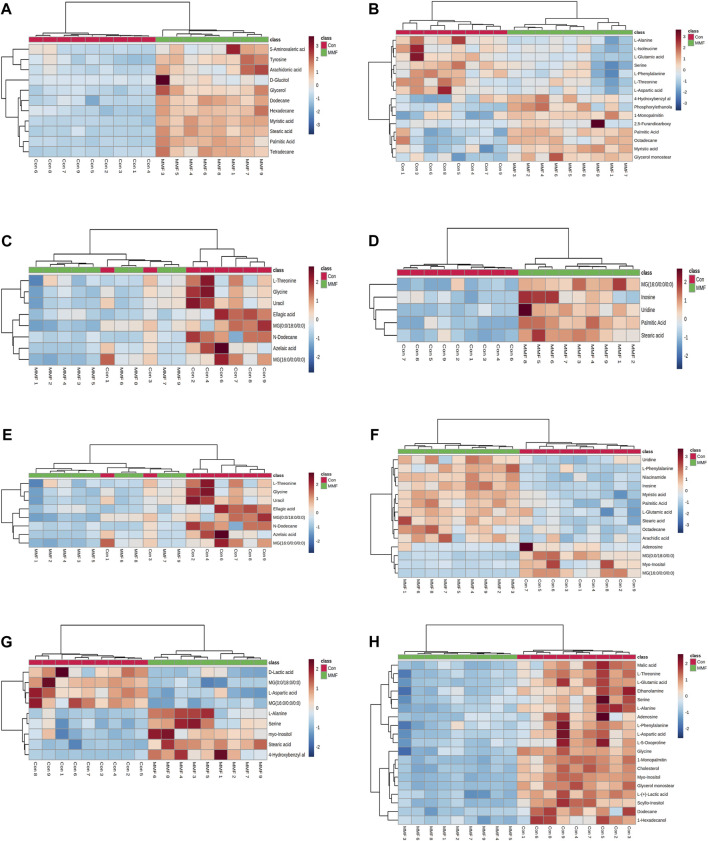
Heatmap of differential metabolites in **(A)** Intestine **(B)** Hippocampus **(C)** Lung **(D)** Liver **(E)** Kidney **(F)** Heart **(G)** Serum **(H)** Cortex in the mycophenolate mofetil (MMF) groups compared with controls. The color of each part represents the importance of metabolite changes (blue, downregulated; red, upregulated). Rows represent samples, and columns represent metabolites.

The differential metabolites in different tissues and serum samples after MMF treatment were exhibited in Table 2. VIP value > 1 and at the same time *p*-value < 0.05 were considered as the important criteria for potential metabolites. Additionally, the values of fold-change (<1) represented that the metabolite levels appeared decreasing trend, whilst those (>1) showed a rising trend. As a results, we found that there was a total of 11 differential metabolites existed in intestine tissues. All of them were upregulated. In hippocampus tissues, 15 differential metabolites were identified. Among these metabolites, 7 metabolites (L-Isoleucine, Serine, L-Threonine, L-Aspartic acid, L-Phenylalanine, L-Alanine and L-Glutamic acid) were observed to be downregulated, while 8 metabolites [O-Phosphoethanolamine, Myristic acid, Palmitic acid, MG(0:0/18:0/0:0), 4-Hydroxybenzyl alcohol, Octadecane, 2,5-Furandicarboxylic acid and MG(16:0/0:0/0:0)] were upregulated. Moreover, we observed MMF treatment altered a total of 8 metabolites in lung tissues [L-Threonine, Glycine, Uracil, MG(0:0/18:0/0:0), N-Dodecane, Ellagic acid, Azelaic acid and MG(16:0/0:0/0:0)], with a downward trend. Only 5 upward metabolites [Palmitic acid, Stearic acid, Uridine, Inosine and MG(16:0/0:0/0:0)] were observed in liver tissues. In the kidney, 12 altered metabolites including 6 downregulated (L-Alanine, Glycine, Glyoxylic acid, Maleic acid, Inosine and Cholesterol) and 6 upregulated metabolites [Myristic acid, L-Tyrosine, O-Phosphoethanolamine, Palmitic acid, MG(16:0/0:0/0:0) and MG(0:0/18:0/0:0)] were identified following MMF treatment. Additionally, in heart samples, a total of 14 altered metabolites were identified, including 4 downregulated [myo-Inositol, MG(16:0/0:0/0:0), Adenosine and MG(0:0/18:0/0:0)] and 10 upregulated metabolites (Niacinamide, L-Glutamic acid, Octadecane, Myristic acid, Palmitic acid, Stearic acid, Arachidic acid, Uridine, L-Phenylalanine and Inosine). In serum samples, 4 downregulated [D-Lactic acid, L-Aspartic acid, MG(16:0/0:0/0:0) and MG(0:0/18:0/0:0)] and 5 upregulated (Serine, Stearic acid, L-Alanine, myo-Inositol and 4-Hydroxybenzyl alcohol) differential metabolites were identified. In cortex samples, a total of 19 downregulated differential metabolites [L-Lactic acid, Ethanolamine, Glycine, Serine, L-Threonine, L-Aspartic acid, L-Phenylalanine, myo-Inositol, L-Alanine, N-Dodecane, Malic acid, Pyroglutamic acid, 1-Hexadecanol, L-Glutamic acid, scyllo-Inositol, MG(16:0/0:0/0:0), Adenosine, MG(0:0/18:0/0:0) and Cholesterol] were identified. As manifested in Figures 3A–H, based on the cluster analyses of differential metabolites between the MMF and control groups, the above results were further validated.

**TABLE 2:** Differential metabolites in different tissues and serum samples after mycophenolate mofetil treatment.

Samples	Metabolites	HMDB ID	VIP	*p*-value	Fold change
Intestine	N-Dodecane	HMDB0031444	1.321	<0.05	2.96
	L-Tyrosine (2TMS)	HMDB0000158	1.261	<0.05	3.36
	Palmitic acid (1TMS)	HMDB0000220	1.414	<0.05	3.40
	Glycerol (3TMS)	HMDB0000131	1.143	<0.05	2.27
	Hexadecane	HMDB0033792	1.278	<0.05	2.82
	Tetradecane	HMDB0059907	1.583	<0.05	5.41
	5-Aminopentanoic acid (3TMS)	HMDB0003355	1.181	<0.05	4.84
	Myristic acid (1TMS)	HMDB0000806	1.547	<0.05	4.71
	Sorbitol (6TMS)	HMDB0000247	1.244	<0.05	5.65
	Stearic acid (1TMS)	HMDB0000827	1.393	<0.05	3.22
	Arachidonic acid (1TMS)	HMDB0001043	1.158	<0.05	3.14
Hippocampus	L-Isoleucine (2TMS)	HMDB0000172	1.090	<0.05	0.64
	Serine (3TMS)	HMDB0062263	1.280	<0.05	0.60
	L-Threonine (2TMS; 3TMS)	HMDB0000167	1.086	<0.05	0.65
	L-Aspartic acid (3TMS)	HMDB0000191	1.273	<0.05	0.60
	L-Phenylalanine (2TMS)	HMDB0000159	1.303	<0.05	0.59
	O-Phosphoethanolamine (4TMS)	HMDB0000224	1.377	<0.05	1.83
	Myristic acid (1TMS)	HMDB0000806	1.489	<0.05	1.83
	Palmitic acid (1TMS)	HMDB0000220	1.206	<0.05	1.47
	MG(0:0/18:0/0:0) (2TMS)	HMDB0011535	1.684	<0.05	2.28
	4-Hydroxybenzyl alcohol (2TBDMS)	HMDB0011724	1.105	<0.05	1.54
	L-Alanine (2TMS)	HMDB0000161	1.564	<0.05	0.49
	L-Glutamic acid (3TMS)	HMDB0000148	1.552	<0.05	0.48
	Octadecane	HMDB0033721	1.227	<0.05	1.64
	2,5-Furandicarboxylic acid (2TMS)	HMDB0004812	1.143	<0.05	2.01
	MG(16:0/0:0/0:0) (2TMS)	HMDB0011564	1.668	<0.05	2.36
Lung	L-Threonine (2TMS)	HMDB0000167	1.086	<0.05	0.75
	Glycine (3TMS)	HMDB0000123	1.091	<0.05	0.62
	Uracil (2TMS)	HMDB0000300	1.173	<0.05	0.58
	MG(0:0/18:0/0:0) (2TMS)	HMDB0011535	1.307	<0.05	0.53
	N-Dodecane	HMDB0031444	1.118	<0.05	0.61
	Ellagic acid	HMDB0002899	1.574	<0.05	0.31
	Azelaic acid (2TBDMS)	HMDB0000784	1.731	<0.05	0.32
	MG(16:0/0:0/0:0) (2TMS)	HMDB0011564	1.476	<0.05	0.47
Liver	Palmitic acid (1TMS)	HMDB0000220	1.483	<0.05	1.78
	Stearic acid (1TMS)	HMDB0000827	1.464	<0.05	1.79
	Uridine (3TMS)	HMDB0000296	2.228	<0.05	5.06
	Inosine (4TMS)	HMDB0000195	1.540	<0.05	2.73
	MG(16:0/0:0/0:0) (2TMS)	HMDB0011564	2.154	<0.05	4.43
Kidney	L-Alanine (2TMS)	HMDB0000161	1.377	<0.05	0.38
	Glycine (2TMS; 3TMS)	HMDB0000123	2.223	<0.05	0.34
	Myristic acid (1TMS)	HMDB0000806	1.392	<0.05	2.24
	L-Tyrosine (2TMS)	HMDB0000158	1.064	<0.05	1.71
	Glyoxylic acid (1TMS)	HMDB0000119	1.241	<0.05	0.53
	Maleic acid (2TMS)	HMDB0000176	1.077	<0.05	0.48
	O-Phosphoethanolamine (4TMS)	HMDB0000224	1.287	<0.05	2.47
	Palmitic acid (1TMS)	HMDB0000220	1.105	<0.05	1.74
	Inosine (4TMS)	HMDB0000195	1.321	<0.05	0.37
	MG(16:0/0:0/0:0) (2TMS)	HMDB0011564	2.309	<0.05	40.38
	MG(0:0/18:0/0:0) (2TMS)	HMDB0011535	2.088	<0.05	10.47
	Cholesterol (1TMS)	HMDB0000067	1.047	<0.05	0.56
Heart	Niacinamide(1TBDMS)	HMDB0001406	1.961	<0.05	3.82
	L-Glutamic acid (3TMS)	HMDB0000148	1.287	<0.05	2.01
	Octadecane (1TMS)	HMDB0033721	1.450	<0.05	2.45
	Myristic acid (1TMS)	HMDB0000806	1.525	<0.05	2.33
	Palmitic acid (1TMS)	HMDB0000220	1.086	<0.05	1.58
	Stearic acid (1TMS)	HMDB0000827	1.178	<0.05	1.69
	Arachidic acid (1TMS)	HMDB0002212	1.245	<0.05	2.06
	Uridine (3TMS)	HMDB0000296	1.423	<0.05	2.22
	L-Phenylalanine (2TMS)	HMDB0000159	1.888	<0.05	4.58
	myo-Inositol (6TMS)	HMDB0000211	1.131	<0.05	0.59
	Inosine (4TMS)	HMDB0000195	2.091	<0.05	5.27
	MG(16:0/0:0/0:0) (2TMS)	HMDB0011564	1.852	<0.05	0.28
	Adenosine (4TMS)	HMDB0000050	1.412	<0.05	0.28
	MG(0:0/18:0/0:0) (2TMS)	HMDB0011535	1.744	<0.05	0.32
Serum	D-Lactic acid (2TMS)	HMDB0001311	1.108	<0.05	0.52
	Serine (3TMS)	HMDB0062263	1.330	<0.05	2.70
	L-Aspartic acid (3TMS)	HMDB0000191	1.309	<0.05	0.45
	Stearic acid (1TMS)	HMDB0000827	1.226	<0.05	1.85
	MG(16:0/0:0/0:0) (2TMS)	HMDB0011564	1.180	<0.05	0.54
	L-Alanine (2TMS)	HMDB0000161	1.590	<0.05	4.88
	myo-Inositol (6TMS)	HMDB0000211	1.209	<0.05	2.20
	MG(0:0/18:0/0:0) (2TMS)	HMDB0011535	1.572	<0.05	0.31
	4-Hydroxybenzyl alcohol (2TBDMS)	HMDB0011724	1.573	<0.05	3.83
Cortex	L-Lactic acid (2TMS)	HMDB0000190	1.063	<0.05	0.54
	Ethanolamine (3TMS)	HMDB0000149	1.195	<0.05	0.40
	Glycine (2TMS)	HMDB0000123	1.304	<0.05	0.37
	Serine (3TMS)	HMDB0062263	1.224	<0.05	0.34
	L-Threonine (3TMS)	HMDB0000167	1.316	<0.05	0.34
	L-Aspartic acid (3TMS)	HMDB0000191	1.322	<0.05	0.30
	L-Phenylalanine (2TMS)	HMDB0000159	1.092	<0.05	0.43
	myo-Inositol (6TMS)	HMDB0000211	1.349	<0.05	0.36
	L-Alanine (2TMS)	HMDB0000161	1.529	<0.05	0.19
	N-Dodecane	HMDB0031444	1.079	<0.05	0.48
	Malic acid (3TMS)	HMDB0000744	1.163	<0.05	0.43
	Pyroglutamic acid (2TMS)	HMDB0000267	1.002	<0.05	0.51
	1-Hexadecanol (1TMS; 1TBDMS)	HMDB0003424	1.234	<0.05	0.36
	L-Glutamic acid (3TMS)	HMDB0000148	1.478	<0.05	0.23
	scyllo-Inositol (6TMS)	HMDB0006088	1.413	<0.05	0.28
	MG(16:0/0:0/0:0) (2TMS)	HMDB0011564	1.480	<0.05	0.27
	Adenosine (4TMS)	HMDB0000050	1.292	<0.05	0.23
	MG(0:0/18:0/0:0) (2TMS)	HMDB0011535	1.461	<0.05	0.28
	Cholesterol (1TMS)	HMDB0000067	1.295	<0.05	0.39

TMS derivative: Trimethylsilyl derivative.

TBDMS derivative: Ter-tbutyldimethylsilyl derivative.

### Metabolic pathway enrichment analysis

**FIGURE 4:**
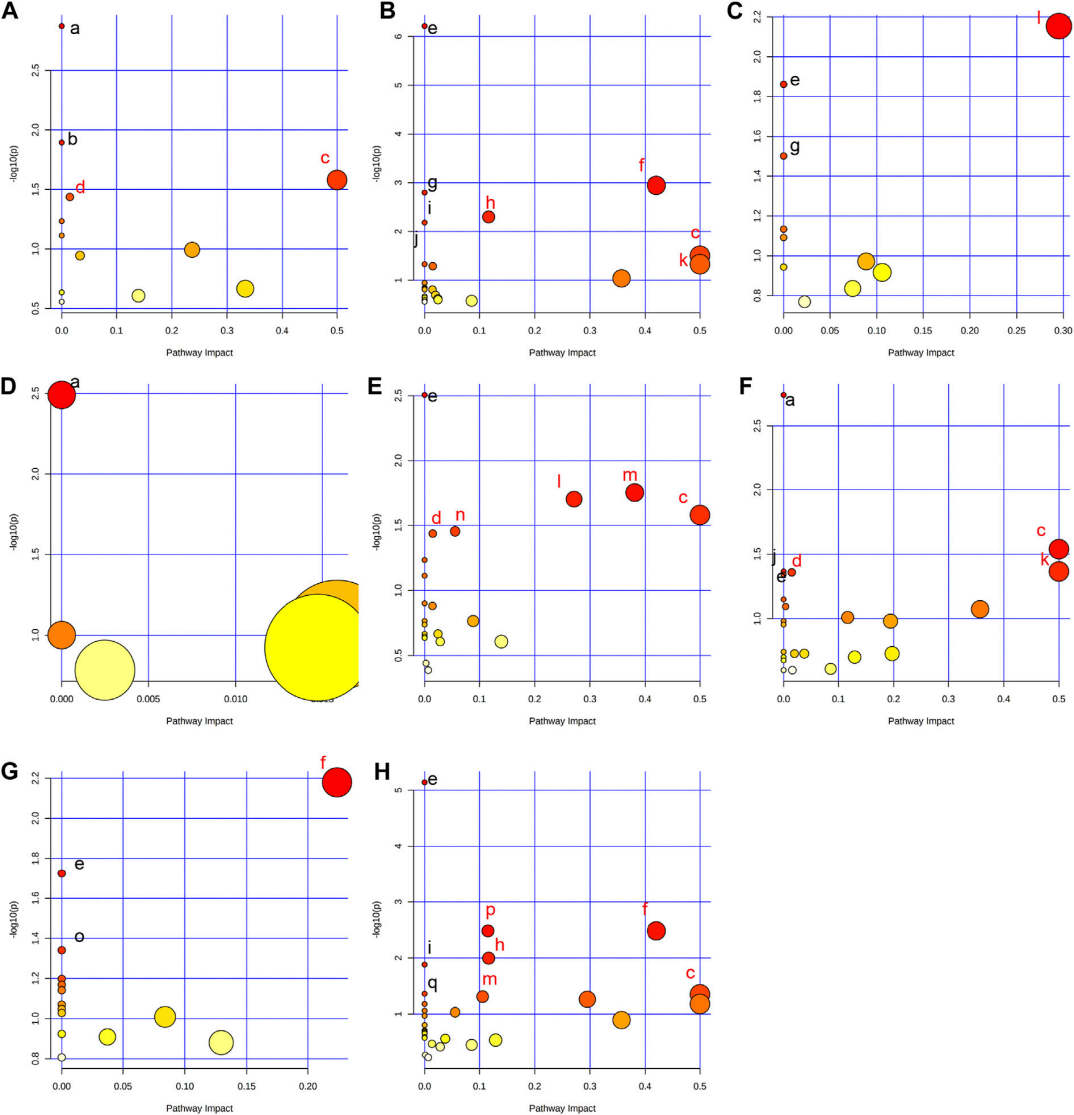
Summary of pathway analysis performed using MetaboAnalyst 5.0. **(A)** Intestine: (a) Aminoacyl-tRNA biosynthesis; (b) Galactose metabolism; (c) Phenylalanine, tyrosine and tryptophan biosynthesis; (d) Fatty acid biosynthesis. **(B)** Hippocampus: (c) Phenylalanine, tyrosine and tryptophan biosynthesis; (e) Aminoacyl-tRNA biosynthesis; (f) Alanine, aspartate and glutamate metabolism; (g) Valine, leucine and isoleucine biosynthesis; (h) Arginine biosynthesis; (i) Histidine metabolism; (j) Nitrogen metabolism; (k) D-Glutamine and D-glutamate metabolism; **(C)** Lung: (e) Aminoacyl-tRNA biosynthesis; (g) Valine, leucine and isoleucine biosynthesis; (l) Glycine, serine and threonine metabolism. **(D)** Liver: (a) Biosynthesis of unsaturated fatty acids. **(E)** Kidney: (c) Phenylalanine, tyrosine and tryptophan biosynthesis; (d) Fatty acid biosynthesis; (e) Aminoacyl-tRNA biosynthesis; (l) Glycine, serine and threonine metabolism; (m) Glyoxylate and dicarboxylate metabolism; (n) Primary bile acid biosynthesis. **(F)** Heart: (a) Biosynthesis of unsaturated fatty acids; (c) Phenylalanine, tyrosine and tryptophan biosynthesis; (d) Fatty acid biosynthesis; (e) Aminoacyl-tRNA biosynthesis; (j) Nitrogen metabolism; (k) D-Glutamine and D-glutamate metabolism. **(G)** Serum: (e) Aminoacyl-tRNA biosynthesis; (f) Alanine, aspartate and glutamate metabolism; (o) Ascorbate and aldarate metabolism. **(H)** Cortex: (c) Phenylalanine, tyrosine and tryptophan biosynthesis; (e) Aminoacyl-tRNA biosynthesis; (f) Alanine, aspartate and glutamate metabolism; (h) Arginine biosynthesis; (i) Histidine metabolism; (m) Glyoxylate and dicarboxylate metabolism; (p) Glutathione metabolism; (q) Porphyrin and chlorophyll metabolism. Black font represents pathways with *p*-value < 0.05 and impact = 0. Red font represents pathways with *p*-value < 0.05 and impact > 0. No labeling font represents pathways with *p*-value > 0.05.

**FIGURE 5:**
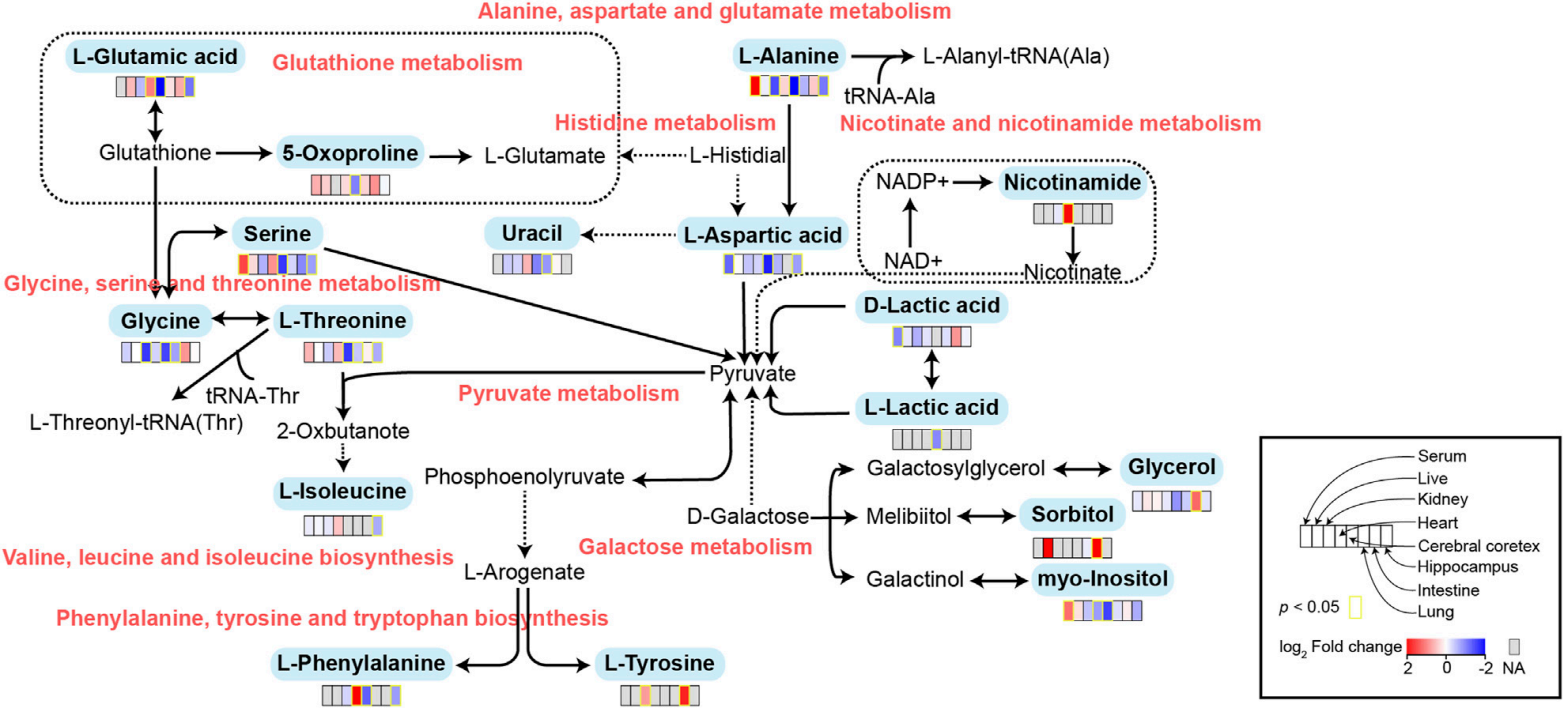
Schematic diagram of related metabolic pathways affected by MMF in serum and major tissues. The activation metabolic pathways were marked in red box. Solid arrows represent a single process, while dashed arrows represent multiple processes. Differential metabolites enriched in pathways were marked in bold.

In order to comprehensively understand the dynamic adaptation of metabolism to MMF treatment, pathway enrichment analysis was performed based on differential metabolites at different tissues. We used Metaboanalyst 5.0 (http://www.metaboanalyst.ca) and KEGG database (http://www.kegg.jp) to analyze the altered metabolic pathways following MMF treatment. Metabolic pathways that the data of Raw *p* less than 0.05 and Impact more than 0 were considered as potential disturbed pathways (Figure 4; Table 3). A detailed metabolic network was presented in Figure 5. We found that MMF challenge not only affected several kinds of amino acid metabolic pathways, but also Phenylalanine, tyrosine and tryptophan biosynthesis; Valine, leucine and isoleucine biosynthesis; Galactose metabolism.

**TABLE 3:** Pathway analysis performed using MetaboAnalyst 5.0 software.

Samples	Pathway name	*p*	Holm *p*	FDR	Impact
Intestine	Phenylalanine, tyrosine and tryptophan biosynthesis	0.03	1	0.737	0.50
	Fatty acid biosynthesis	0.04	1	0.768	0.01
Hippocampus	Alanine, aspartate and glutamate metabolism	0.00	0.094055	0.044	0.42
	Arginine biosynthesis	0.01	0.407	0.106	0.12
	Phenylalanine, tyrosine and tryptophan biosynthesis	0.03	1	0.441	0.50
	D-Glutamine and D-glutamate metabolism	0.05	1	0.481	0.50
Lung	Glycine, serine and threonine metabolism	0.01	0.58916	0.57762	0.30
Kidney	Glyoxylate and dicarboxylate metabolism	0.02	1	0.512	0.38
	Glycine, serine and threonine metabolism	0.02	1	0.512	0.27
	Phenylalanine, tyrosine and tryptophan biosynthesis	0.03	1	0.512	0.50
	Primary bile acid biosynthesis	0.04	1	0.512	0.06
	Fatty acid biosynthesis	0.04	1	0.512	0.01
Heart	Phenylalanine, tyrosine and tryptophan biosynthesis	0.03	1	0.63774	0.50
	D-Glutamine and D-glutamate metabolism	0.04	1	0.63774	0.50
	Fatty acid biosynthesis	0.04	1	0.63774	0.01
Serum	Alanine, aspartate and glutamate metabolism	0.01	0.55531	0.55531	0.22
Cortex	Glutathione metabolism	0.00	0.27309	0.092	0.12
	Alanine, aspartate and glutamate metabolism	0.00	0.27309	0.092	0.42
	Arginine biosynthesis	0.01	0.81665	0.212	0.12
	Phenylalanine, tyrosine and tryptophan biosynthesis	0.04	1	0.504	0.50
	Glyoxylate and dicarboxylate metabolism	0.05	1	0.504	0.11

### Multiparameter flow cytometry analysis for lymphocytes

**FIGURE 6:**
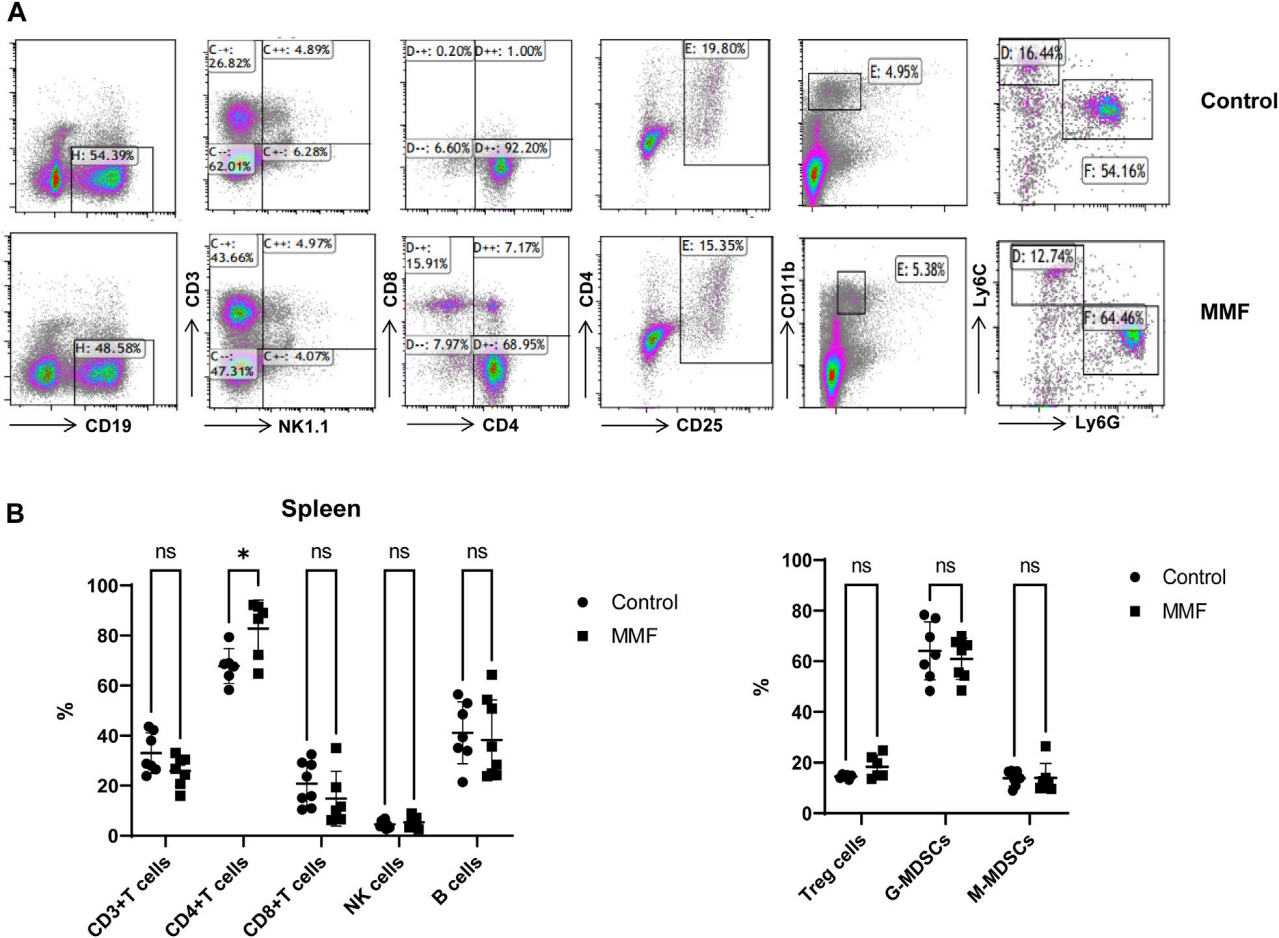
Multiparameter flow cytometry analysis for the percentages of lymphocytes from spleen. **(A)** Representative images of multiparameter flow cytometry analysis. **(B)** The percentages of CD3^+^ T cells, CD4^+^ T cells, CD8^+^ T cells, Natural Killer (NK) cells, B cells, Treg cells, peripheral granulocytic-myeloid-derived suppressor cells (G-MDSCs) and monocytic-myeloid-derived suppressor cells (M-MDSCs) in the control and MMF groups. *p < 0.05. ns, no significance.

Spleen contains a large number of lymphocytes, which plays an important role in the immune processes. Therefore, we isolated lymphocytes from the spleen tissues of mice in different groups to validate the immunosuppressive mechanism of MMF. The percentages of CD3^+^ T cells, CD4^+^ T cells, CD8^+^ T cells, Natural Killer (NK) cells, B cells, peripheral granulocytic-myeloid-derived suppressor cells (G-MDSCs), monocytic-myeloid-derived suppressor cells (M-MDSCs) and Treg were calculated. The results indicated that MMF administration dramatically increased CD4^+^ T cell percentages (*p*-value < 0.05) but had no significant influences on other types of lymphocytes (Figure 6).

## Discussion

MMF is a kind of new immunosuppressive agents, which can prevent the replication of T and B lymphocytes by inhibiting purine synthesis (Allison and Eugui, 1993). The usage of MMF is generally associated with gastrointestinal side effects and among these incidental consequences, treatment of MMF has been confirmed to cause the disorder of several metabolic pathways such as glucuronide/glucoside, plasma bioelement contents, and nucleotide and lipid metabolism (Shipkova et al., 2005; Kaminska et al., 2012; Heischmann et al., 2017). However, the metabolic profiling of each organ following MMF treatment is still poorly understood.

In the current work, a GC-MS approach was utilized to ascertain the alteration of metabolite in serum and tissue samples (intestine, hippocampus, lung, liver, kidney, heart, cortex) of MMF-challenged mice. MMF can influence various organs through diverse pathways, encompassing variations in drug metabolism, organ-specific physiological responses, and heterogeneous drug distribution. Distinct metabolic product profiles may emerge in different organs due to differences in drug metabolism pathways. Moreover, the unique physiological functions and metabolic characteristics of each organ may yield diverse metabolic effects. Additionally, the non-uniform distribution of the drug in the body can result in varying organ exposures to different drug concentrations, contributing to differences in metabolic levels (Liu et al., 2022). This can clarify why MMF has a completely different metabolic output on various organs. Additionally, based on the pathway analysis results via MetaboAnalyst 5.0 software, we found that several amino acid metabolism-related pathways including alanine, aspartate and glutamate metabolism, arginine biosynthesis, histidine metabolism, and phenylalanine, tyrosine and tryptophan biosynthesis were remarkably disrupted in hippocampus and cortex. Specially, the alteration of nitrogen metabolism and D-Glutamine and D-glutamate metabolism in hippocampus, and glutathione metabolism, porphyrin and chlorophyll metabolism, and glyoxylate and dicarboxylate metabolism in cortex were observed. Some previous researches have demonstrated that the onset of seizures and encephalopathy may be related to the use of MMF in transplant patients (Derle et al., 2015; Pellerin et al., 2018). Generally, lipophilic antibiotics can reach the infection compartment through blood-brain barrier and it is well-known that MMF is indeed lipophilic (Iqbal et al., 2020). Therefore, we speculated that MMF may possess neurotoxicity via affecting the metabolism disorders in hippocampus and cortex.

Amino acids, as important substrates, play a regulatory role in many metabolic pathways and function as diagnostic markers of many diseases (Razak et al., 2017). Several kinds of amino acids including glycine, serine, L-isoleucine, L-threonine, L-aspartic acid, L-phenylalanine, L-alanine, and L-glutamic acid were downregulated in MMF group compared to those of control group. Glycine has very vital roles in cytoprotection, immune response, growth, development, metabolism, and survival of many mammals and humans. In central nervous system, glycine is considered as an important neurotransmitter to control food intake and body homeostasis (Rajendra et al., 1997) and at the same time, it can regulate the immune function, superoxide generation and cytokines synthesis by modulating the levels of intracellular Ca^2+^ (Zhong et al., 2003). Additionally, we also found that excessive fatty acids such as palmitic acid, myristic acid, stearic acid were existed in the intestine, hippocampus, liver, kidney, heart tissues and serum samples of rats following MMF treatment, while high levels of fatty acids have been confirmed to inhibit the amount of glycine (Dasarathy et al., 2009). We speculated that MMF administration may not only induce neurotoxicity but also cause toxicity in other organs.

To further explore the mechanism of MMF-induced GI toxicity, we found the disorder of phenylalanine, tyrosine and tryptophan biosynthesis, and fatty acid biosynthesis in intestine tissues. Apart from the effects of the disruption of these two metabolic pathways on intestine metabolism, we further indicated that glycerol and sorbitol that involved in galactose metabolism were two main differential metabolites in intestine tissues, indicating that MMF treatment also disrupted galactose metabolism, as shown in Figure 5. Lactose is a disaccharide of galactose and glucose that is hydrolyzed to monosaccharides by enterocytes. Galactose can be absorbed by the mature enterocytes at the tips of the villi (Wright, 2013). Generally, galactose metabolism can be catalyzed sequentially by three enzymatic steps, with the aid of enzymes galactokinase, UTP-hexose-1-phosphate uridylyltransferase and UDP-galactose 4′-epimerase, respectively (Coelho et al., 2015). At the same time, immunosuppression can universally repress the activity of UDP-galactose 4′-epimerase (Lee et al., 2014), while deficiency of each one of the three galactose-metabolism-related enzymes can lead to galactosemias, such as *Escherichia coli* sepsis (Novelli and Reichardt, 2000). Because MMF is widely used for maintenance immunosuppression in solid organ, bone marrow, and stem cell transplantation (Srinivas et al., 2003b; Moiseev et al., 2016b; Carlone et al., 2021b), we therefore speculated that MMF treatment may induce intestine toxicity via immunosuppression. Additionally, it is reported that after efflux from the enterocyte, approximately 88% of the ingested galactose is retained in the liver, while the remaining amounts are transported into brain for amino acid biosynthesis (Roser et al., 2009; Augustin, 2010; Chichlowski et al., 2011). Therefore, the disorder of galactose metabolism may also affect the biosynthesis of amino acids in brains, which may further validate the neurotoxicity of MMF administration.

Glutathione (GSH) is a crucial endogenous antioxidant and glutamate serving as a precursor to exert important functions for GSH generation (Mothet et al., 2005). The disorder of D-Glutamine and D-glutamate metabolism was not only observed in hippocampus, but also in the heart tissues. Meanwhile, we also demonstrated that glutathione metabolism was disrupted in cortex. These results implied that following treatment of MMF, the imbalance of redox may be occurred in hippocampus, heart and cortex. Nitrogen is essential for the synthesis of a variety of biomolecules including amino acids, porphyrins, nucleotides, glutathione and other critical biological compounds. Glutamine and glutamate metabolism are central to nitrogen metabolism, and low levels of glutamine and glutamate can limit the flow of nitrogen within cells and delay biomass production and growth (Kurmi and Haigis, 2020). Therefore, it is speculated that MMF administration may first disrupt the metabolism of glutamine and glutamate and then nitrogen metabolism, eventually disturbing the balance of amino acid biosynthesis, glutathione metabolism and porphyrin and chlorophyll metabolism in multiple organs. Additionally, some researches have uncovered that the absence of tryptophan can promote immune suppression and at the same time, arginine is confirmed to exert vital role in T cell activation, especially the survival of CD4^+^ T cells (O’Sullivan et al., 2019; Geiger et al., 2016). In our study, we found that phenylalanine, tyrosine and tryptophan biosynthesis was disrupted in multiple organs including intestine, hippocampus, kidney, heart and cortex, while the disorder of arginine biosynthesis was observed in hippocampus and cortex. We hypothesized that MMF treatment may disrupt phenylalanine, tyrosine and tryptophan biosynthesis and arginine biosynthesis to exert immunosuppression functions. Additionally, the percentages of CD3^+^ T cells, CD4^+^ T cells, CD8^+^ T cells, NK cells, B cells, MDSCs and Treg were also calculated. A report conducted by Sousa et al. has demonstrated that the depletion of CD4^+^ T cells is directly associated with the activation of immune system (Sousa et al., 2002). In the current study, the results indicated that MMF administration dramatically increased CD4^+^ T cell percentages but had no significant influences on other types of lymphocytes, indicating that the increased percentages of CD4^+^ T cells may prohibit immune system. These results also elucidated the immunosuppressive mechanism of MMF, to some extent.

Some limitations in this work should not be ignored. First, only a simplex metabonomic analysis was employed in this study and more analytical approaches may be needed. Second, performing GC-MS metabolomics on CD4^+^ T cells and CD8^+^ T cells is beneficial to further shed light on the potential mechanism of action of MMF on immune cells.

## Conclusion

In a word, the current study analyzed the metabolic profiling of serum samples and organ tissues in mice following MMF treatment, indicating that MMF challenge may not only induce neurotoxicity in hippocampus and cortex, but also cause toxicity in other organs. More importantly, MMF administration may exert immunosuppressive functions via decreasing CD4^+^ T cell percentages. These findings provide preliminary insights into metabolomic identification after MMF administration.

## Data Availability

The raw data supporting the conclusion of this article will be made available by the authors, without undue reservation.

## Abbreviations

MMF, Mycophenolate mofetil; GC-MS, Gas chromatography-mass spectrometry; DMSO, dimethyl sulfoxide; KEGG, Kyoto Encyclopedia of Genes and Genomes; MPA, mycophenolic acid; GI, gastrointestinal; LPS, lipopolysaccharide; QC, quality control; TICs, total ion chromatograms; PCA, principal components analysis; OPLS-DA, orthogonal partial least squares discrimination analysis; NK, Natural Killer; G-MDSCs, granulocytic-myeloid-derived suppressor cells; M-MDSCs, monocytic-myeloid-derived suppressor cells; GSH, Glutathione.
