# Supplementary figures and images for: A GC–MS-based untargeted metabolomics approach for comprehensive metabolic profiling of mycophenolate mofetil-induced toxicity in mice
Source: Front Mol Biosci. 2024 Mar 7;11:1332090. doi: 10.3389/fmolb.2024.1332090 (PMC10955473; doi:10.3389/fmolb.2024.1332090)

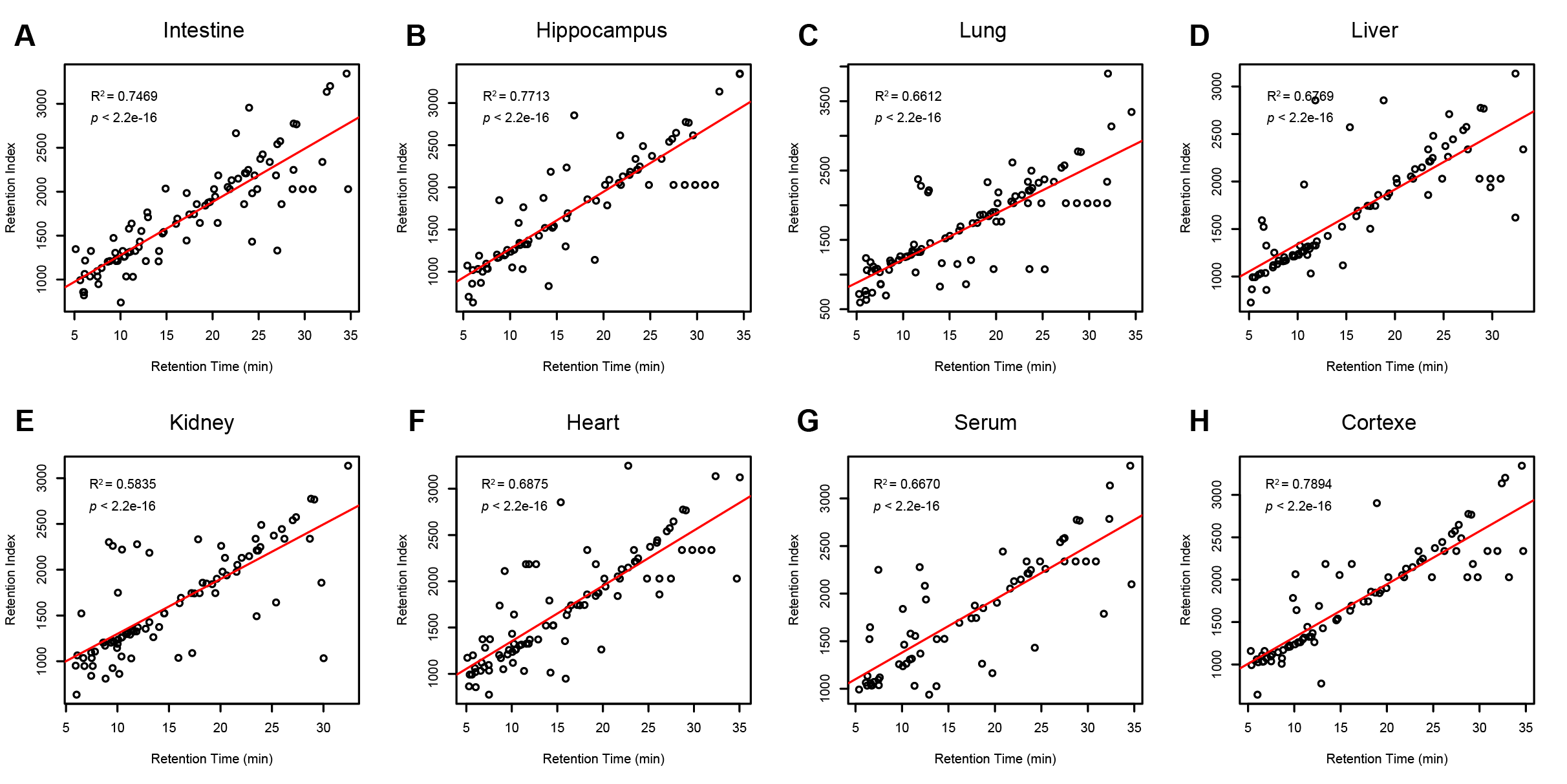

Supplement: Supplementary file 1 [file Image1.TIF]
